# Zygotic Vsx1 Plays a Key Role in Defining V2a Interneuron Sub-Lineage by Directly Repressing *tal1* Transcription in Zebrafish

**DOI:** 10.3390/ijms21103600

**Published:** 2020-05-20

**Authors:** Qi Zhang, Haomang Xu, Wei Zhao, Jianbo Zheng, Lei Sun, Chen Luo

**Affiliations:** 1 College of Life Sciences, Zhejiang University, Hangzhou 310058, China; zhangqi5400@163.com (Q.Z.); haomang@zju.edu.cn (H.X.); zhaowei086@zju.edu.cn (W.Z.); 21207054@zju.edu.cn (J.Z.); 10907031@zju.edu.cn (L.S.); 2 Zhejiang Institute of Freshwater Fisheries, Huzhou 313001, China

**Keywords:** V2 interneuron diversification, V2a interneuron specification, Vsx1, Tal1, transcriptional repression, spinal cord, zebrafish

## Abstract

In the spinal cord, excitatory V2a and inhibitory V2b interneurons are produced together by the final division of common P2 progenitors. During V2a and V2b diversification, Tal1 is necessary and sufficient to promote V2b differentiation and Vsx2 suppresses the expression of motor neuron genes to consolidate V2a interneuron identity. The expression program of Tal1 is triggered by a Foxn4-driven regulatory network in the common P2 progenitors. Why the expression of Tal1 is inhibited in V2a interneurons at the onset of V2a and V2b sub-lineage diversification remains unclear. Since transcription repressor Vsx1 is expressed in the P2 progenitors and newborn V2a cells in zebrafish, we investigated the role of Vsx1 in V2a fate specification during V2a and V2b interneuron diversification in this species by loss and gain-of-function experiments. In *vsx1* knockdown embryos or knockout Go chimeric embryos, *tal1* was ectopically expressed in the presumptive V2a cells, while the generation of V2a interneurons was significantly suppressed. By contrast, in *vsx1* overexpression embryos, normal expression of *tal1* in the presumptive V2b cells was suppressed, while the generation of V2a interneuron was expanded. Chromatin immunoprecipitation and electrophoretic mobility shift assays in combination with core consensus sequence mutation analysis further revealed that Vsx1 can directly bind to *tal1* promoter and repress *tal1* transcription. These results indicate that Vsx1 can directly repress *tal1* transcription and plays an essential role in defining V2a interneuron sub-lineage during V2a and V2b sub-lineage diversification in zebrafish.

## 1. Introduction

In the spinal cord, excitatory V2a interneurons are crucial to the transmission and coordination of motor function [1,2,3,4,5]. It is well known that excitatory V2a interneurons are generated together with inhibitory V2b interneurons by the final division of common P2 progenitors [6,7,8,9,10,11,12]. Many regulatory factors involved in both V2a and V2b interneuron development have been identified in vertebrates [7,11,13,14,15,16,17,18]. During the diversification of V2a and V2b interneurons, Tal1 (also called Scl) is necessary and sufficient to promote V2b interneuron differentiation [10,13,15], and Vsx2 (also called Chx10) suppresses the expression of motor neuron genes to consolidate V2a interneuron identity [16]. Many studies have revealed that both Tal1 and Vsx2 expression programs are triggered in the common V2 precursors. Foxn4 promotes a complex regulatory network, including Gata3, Mash1 and Delta ligands, and activates Notch-delta signaling in the P2 progenitors [7,14]. After the final division of common P2 progenitors, Gata3 and the activated Notch-delta signaling in turn jointly promote *tal1* expression in one of the P2 daughter cells to define V2b sub-lineage [7,10,11,14]. Lhx3 is able to trigger Vsx2 expression and V2a interneuron development program in the P2 progenitors. The misexpression of Lhx3 can induce ectopic expression of Vsx2 and drive ectopic formation of V2a interneurons in the developing spinal cord [17,18]. However, it remains unclear why the expression of Tal1 is inhibited in the presumptive V2a cells at the onset of V2a and V2b diversification.

A paired-like transcription factor Vsx1, containing an evolutionary conservative homeodomain and CVC domain, is the paralog of Vsx2 [19,20,21,22]. Previous studies have demonstrated that Vsx1 can directly repress target genes expression [23,24]. In all the examined vertebrates, Vsx1 is expressed in the P2 precursors, while Vsx2 is expressed in mature V2a cells [11,12,19,20,21,22]. After the final mitotic division of V2 precursors, the expression of Vsx1 is maintained in V2a but not in V2b cells at the early diversification stage in zebrafish [11,12]. Interestingly, in the zebrafish Notch signaling mutant, the loss of *tal1* expression is associated with ectopic expression of Vsx1 in V2b cells [10,11]. These observations stimulate us to investigate whether Vsx1 plays a role in V2a fate specification by inhibiting Tal1 expression in zebrafish. Our experiments show that zygotic Vsx1 can directly repress *tal1* transcription and is essential for protecting V2a interneuron fate specification during V2a and V2b sub-lineages diversification in zebrafish.

## 2. Results

### 2.1. Zygotic Vsx1 Has no Impact on the Generation of both P2 Progenitors and Its Adjacent Neurons

In the eggs of zebrafish, there are trace amounts of maternal *vsx1* transcripts [19] that are crucial to the formation of axial mesoderm at the early development stage [23]. Blocking the function of maternal *vsx1* transcripts can cause a loss of axial mesoderm, which in turn severely disturbs spinal cord development [23]. Therefore, we used blocking mRNA splicing in combination with transient directed gene knockout, which can suppress the function of the zygotic *vsx1* gene but avoid damaging the maternal mature *vsx1* mRNA, to examine the role of zygotic Vsx1 in V2a and V2b diversification in the spinal cord.

**Figure 1:**
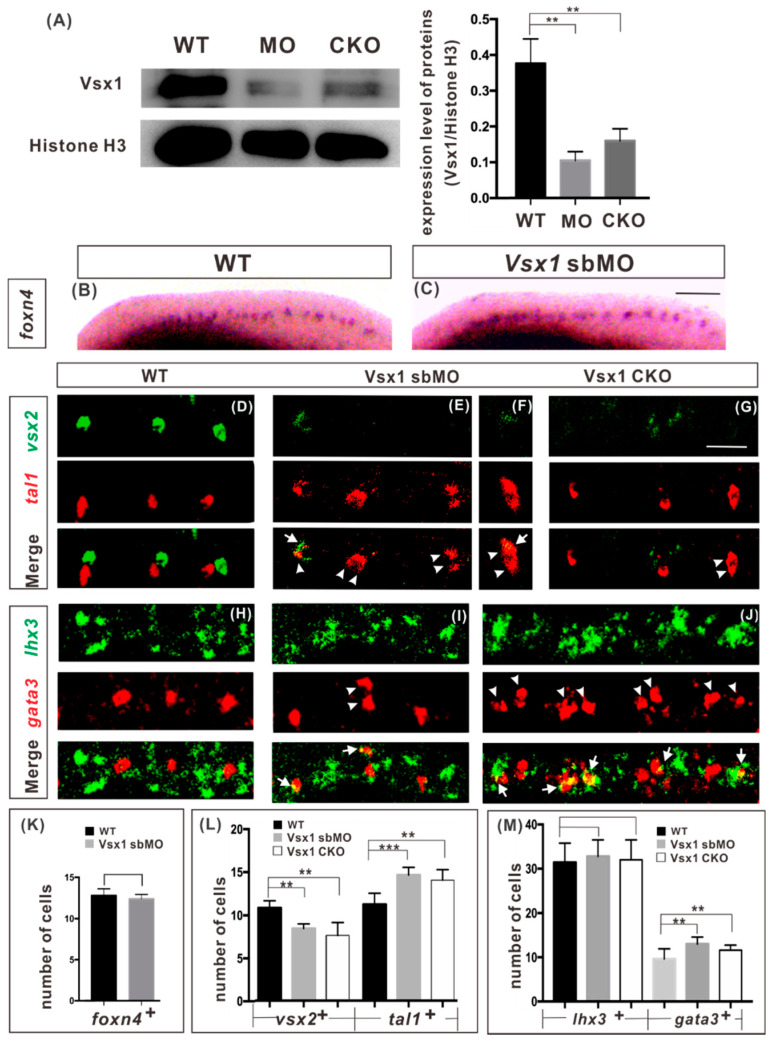
Zygotic Vsx1 is essential for preventing *tal1* expression in presumptive V2a cells. (**A**) Western blot analysis of *vsx1* sbMO knockdown and CRISPR/Cas9 transient knock out efficiency in 24 hpf zebrafish embryo. ** *p* ≤ 0.01. WT: wild type, MO: morpholino, CKO: chimeric knock out. (**B**,**C**) In situ hybridization of P2 progenitor marker *foxn4* in the wide type (**B**) and zygotic *vsx1* knockdown (**C**) embryos at 18 hpf. (**D**–**G**) Fluorescent double in situ hybridization of *vsx2* (green) and *tal1* (red) at 20 hpf in the wild type (**D**), zygotic *vsx1* knockdown (**E**,**F**) and *vsx1* chimeric knockout G0 (**G**) embryos. The expression of *vsx2* was repressed but the expression of *tal1* was detected in two cells (**E**,**F**) in some segments of zygotic *vsx1* knockdown embryos. The same repression of *vsx2* and ectopic expression of *tal1* was detected in *vsx1* chimeric knockout G0 embryos (**G**). (**H**–**J**) Fluorescent double in situ hybridization of *lhx3* (green) and *gata3* (red) at 20 hpf in the wild type (H), zygotic *vsx1* knockdown (**I**) and *vsx1* chimeric knockout G0 (**J**) embryos. *gata3* ectopic expression and *lhx3-gata3* co-expressing cells were detected in zygotic *vsx1* knockdown (**I**) and *vsx1* chimeric knockout embryos (**J**). White arrow heads indicate *tal1*-expressing cell. White arrows indicate *tal1* and *vsx2* co-expressing cells. The injected reagents are indicated at the tops of images and makers are indicated on the left sides of images. Dorsal is upwards; anterior is leftwards. Scale bars: 100 μm in B-C, 12.5 μm in D-J. (**K**–**M**) Quantification of marked neurons number. Counts of *foxn4, vsx2, tal1, lhx3, gata3*-expressing cells were derived from both sides of spinal cord above the yolk extension over a five-somite distance. Data in wild-type (black), sbMO knockdown (gray) and chimeric knockout (white) are presented as means ± SEMs from 10 embryos from at least two independent experiments. Statistical significance was assessed using the unpaired two-tailed Student’s *t*-test. ** *p* ≤ 0.01, *** *p* ≤ 0.001.

A previously used splice-blocking MO (sbMO), which targets to the first splicing site of *vsx1* mRNA and can efficiently block the splicing of newly synthesized zygotic *vsx1* mRNA [24,25], was employed and 15 ng of *vsx1* sbMO was injected into the embryos at one cell stage in our experiment. *vsx1* knockout was done by co-injecting 345 pg target gRNA and 690 pg CRISPR/Cas9 protein at one cell stage. Genome sequence analysis showed that about 70% of the examined knockout G0 embryos (*n* = 30) were chimeric mutants with different *vsx1* mutations (Figure S1). Western blot analysis showed that the level of Vsx1 protein in both *vsx1* sbMO injected embryos and CRISPR/Cas9 protein injected embryos was significantly lower than that in the wild type embryos at 24 hpf (Figure 1A), indicating that Vsx1 synthesis was effectively suppressed in these embryos. Both *vsx1* knockdown and transient directed *vsx1* knockout embryos exhibited no visible morphological and structural deformities in appearance until 27 hpf (Figures S2 and S3). At 72 hpf stage, in 73% of *vsx1* knockdown embryos (*n* = 201, Figure S2C–E) and in 63% of chimeric knockout G0 embryos (*n* = 162, Figure S3D), the head and eyes were significantly smaller, but the yolk sacs were bigger than those in the wild type embryos (Figure S2 and S3). The phenotypes of Vsx1 Cas9 knockout F0 embryos were similar to Vsx1 sbMO knockdown embryos at both 27 and 72 hpf, indicating that *vsx1* sbMO can specifically inhibit the function of zygotic *vsx1*.

During vertebrate development, neural progenitor cells initially group together into distinct domains along the dorsal-ventral axis of the spinal cord in response to signals emanating from local organizing centers, and then the neural progenitor cells in each domain generate distinct neuronal subtypes to establish neuronal circuits [26,27]. Since zygotic *vsx1* is initially expressed in common V2a and V2b progenitor P2 cells in developing neural tube [11,12,19], we first examined whether blocking the function of zygotic Vsx1 affected the generation of P2 progenitors and its adjacent dorsal V1 interneurons and ventral motor neurons. Whole mount in situ hybridization of P2 marker gene *foxn4* expression showed that the number of P2 cells had no detectable difference between the wild-type and sbMO injected embryos (Figure 1B,C,K). Whole mount fluorescent in situ hybridization (WISH) on the expression of V1 interneuron marker *eng1b* and motor neuron marker *isl1* and *2* showed that the numbers of both V1 interneurons and motor neurons in *vsx1* knockdown embryos were indistinguishable to those in the wild-type embryos at 20 hpf (Figure S4). It is clear that inhibition of zygotic Vsx1 has no impact on the generation of P2 progenitor and its adjacent neurons on both dorsal and ventral sides.

### 2.2. Vsx1 is Essential for Repressing Tal1 Expression in Presumptive V2a Cells and Defining V2a Sub-Lineage

To determine the role of zygotic Vsx1 in V2a and V2b diversification, we examined the expression of V2a marker gene *vsx2* and V2b marker gene *tal1* in *vsx1* knockdown and chimeric knockout G0 embryos by fluorescent WISH. At 20 hpf, in wide-type embryos the division of P2 progenitors was initiated, and V2a and V2b neurons were specified by the exclusive detection of Vsx2 and Tal1 [12] (Figure 1D). In zygotic *vsx1* knockdown embryos, the expression of *vsx2* was repressed in different degrees, in conjunction with the ectopic expression of *tal1.* In some segments, the expression of *vsx2* was completely repressed and two *tal1*-expressing cells were detected. In other segments, the expression of *vsx2* was partially repressed and there was a cell with overlap of *vsx2* and *tal1* signals and a normal *tal1*-expressing V2b cell. Further quantitative analysis showed that the number of *vsx2*-positive V2a cells was significantly decreased (by about 27%), while the number of *tal1*-positive V2b interneurons was elevated (by about 30%) in zygotic *vsx1* knockdown embryos, compared to that in wild-type embryos (Figure 1E,F,L). Similar results were observed in chimeric vsx1 knockout G0 embryos (Figure 1G,L), demonstrating that inhibiting the function of zygotic Vsx1 elicited ectopic expression of *tal1* in presumptive V2a cells in zebrafish spinal cords.

A recent study in zebrafish has identified a novel neuronal V2 subtype V2s [28], which shares same markers and regulatory factors (including *tal1*) as V2b cells [28], suggesting that V2s and V2b cells might share a common origin and their fate specifications are regulated initially by the same factors. We further analyzed the number of V2s cells in *vsx1* knockdown embryos at 20 hpf by detecting the expression of V2s markers *sox1a* and *sox1b*. The results showed that the expression level of the V2s markers was unchanged between wild type and *vsx1* knockdown embryos, and the numbers of *tal1* and V2s marker co-expression cells were also at the same levels in both types of embryo (Figure S5). It is clear that blocking the function of Vsx1 has no impact on V2s formation at 20 hpf.

To confirm that V2b interneuron development program was ectopically activated in presumptive V2a cells in Vsx1 deficient embryos, we further examined the expression of *lhx3*, an early V2a cell marker, and *gata3*, a V2b cell marker downstream of *tal1*, in *vsx1* knockdown and chimeric knockout G0 embryos. Unlike *vsx2*, *lhx3* is initially expressed in both P2 progenitors and motor neuron progenitors, and then restricted in V2a and motor neurons but not in V2b cells after the division of the progenitors [9,10,11]. Fluorescent WISH showed that, in both Vsx1 knockdown and chimeric knockout G0 embryos at 20 hpf, the number of *gata3*-expressing V2b cells was significantly increased, but the number of *lhx3*-expressing cells was the same as that observed in the wild type (Figure 1H–J,M). The co-expression of *lhx3* and *gata3* was detected in about 73% of examined *vsx1* knockdown embryos (*n* = 26) and 69% of examined *vsx1* transient knockout embryos (*n* = 29; Figure 1I,J,M). A small number of *lhx3*-positive cells co-expressed *gata3*, about 8.7% (*n* = 334) in *vsx1* knockdown embryos and 9.8% (*n* = 345) in chimeric knockout G0 embryos, respectively. (Figure 1M). Based on the above observation that inhibiting the function of zygotic Vsx1 has no impact on motor neuron generation (Figure S4F), we conclude that the number of *lhx3*-expressing V2 cells was not affected, but some *lhx3*-expressing V2 cells adopted the V2b interneuron development program instead of its presumptive V2a interneuron development program in both types of Vsx1 deficient embryos.

**Figure 2:**
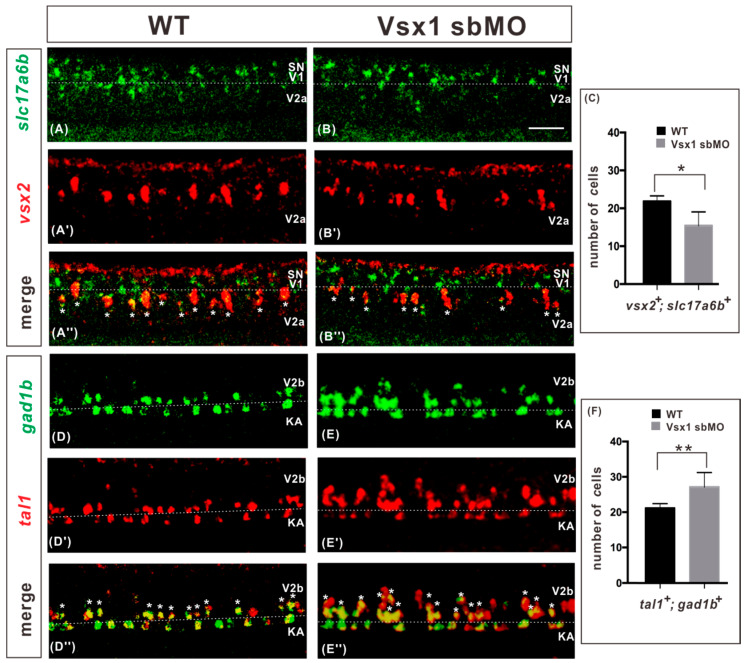
Excitatory V2a and inhibitory V2b interneurons in the wild type and *vsx1* knockdown embryos at 27 hpf. (**A**–**C**) Fluorescent double in situ hybridization of *slc17a6b* (green) and *vsx2* (red) show that the *slc17a6b* and vsx2 co-expressing excitatory V2a cells were decreased by 30% in *vsx1* knockdown spinal cord. (**D**–**F**) Fluorescent double in situ hybridization of *gad1b* (green) and *tal1* (red) show that the *gad1b* and *tal1* co-expressing inhibitory V2b cells were increased by 28% in *vsx1* knockdown spinal cords. White dotted lines indicate the boundaries between adjacent neuron regions. Asterisks indicate double-labeled cells. The injected reagents are indicated at the tops of images and makers are indicated on the left sides of images. Dorsal is upwards; anterior is leftwards. Scale bars: 25 μm. Counts of *slc17a6b*-*vsx2* co-expressing cells and *gad1b*-*tal1* co-expressing cells were derived from somite 6–12 on both spinal cord sides. Data in wild-type (black) and knockdown (gray) are presented as means ± SEMs in C and F from 10 embryos from at least two independent experiments. Statistical significance was assessed using the unpaired two-tailed Student’s *t*-test. * *p* ≤ 0.05, ** *p* ≤ 0.01.

Next, we compared the terminal differentiation of excitatory V2a and inhibitory V2b interneurons between the wild-type and *vsx1* knockdown embryos at 27 hpf. The expressions of *slc17a6b* (also called *vglut2.1*) and *gad1b* (also called *gad67*) were used as the neurotransmitter markers for excitatory V2a and inhibitory V2b interneurons, respectively, in zebrafish [11]. Fluorescent WISH showed that, in the spinal cords of zygotic *vsx1* knockdown embryos, the number of *slc17a6b* and *vsx2* co-expressing excitatory V2a interneurons was decreased by 30% (Figure 2C), almost equal to the 27% reduction in the number of *vsx2*-expressing cells shown in Figure 1L. Meanwhile, the number of *gad1b* and *tal1* co-expressing inhibitory V2b interneurons was increased about 28% (Figure 2F), almost equal to the 30% increase in the number of *tal1*-expressing cells shown in Figure 1L. These results verified that, in association with the decrease of excitatory V2a interneuron number, the number of inhibitory V2b interneuron increased in equal proportion in the spinal cord of zygotic Vsx1 knockdown embryos. Taken together, we conclude that blocking the function of zygotic Vsx1 resulted in ectopic activation of V2b interneuron development program in presumptive V2a cells in the spinal cord.

### 2.3. Vsx1 Overexpression can Repress tal1 Expression in Presumptive V2b Cells and Elicit Ectopic V2a Generation

If Vsx1 can repress *tal1* expression, one can expect that *vsx1* and *tal1* will not be expressed in the same cell. Fluorescent WISH showed that it is indeed the case. In all the examined wild type embryo at 20 hpf, both *vsx1* and *tal1* were expressed in the V2 domain of a spinal cord, but no *vsx1* and *tal1* co-expressing cell was detected (Figure S6).

**Figure 3:**
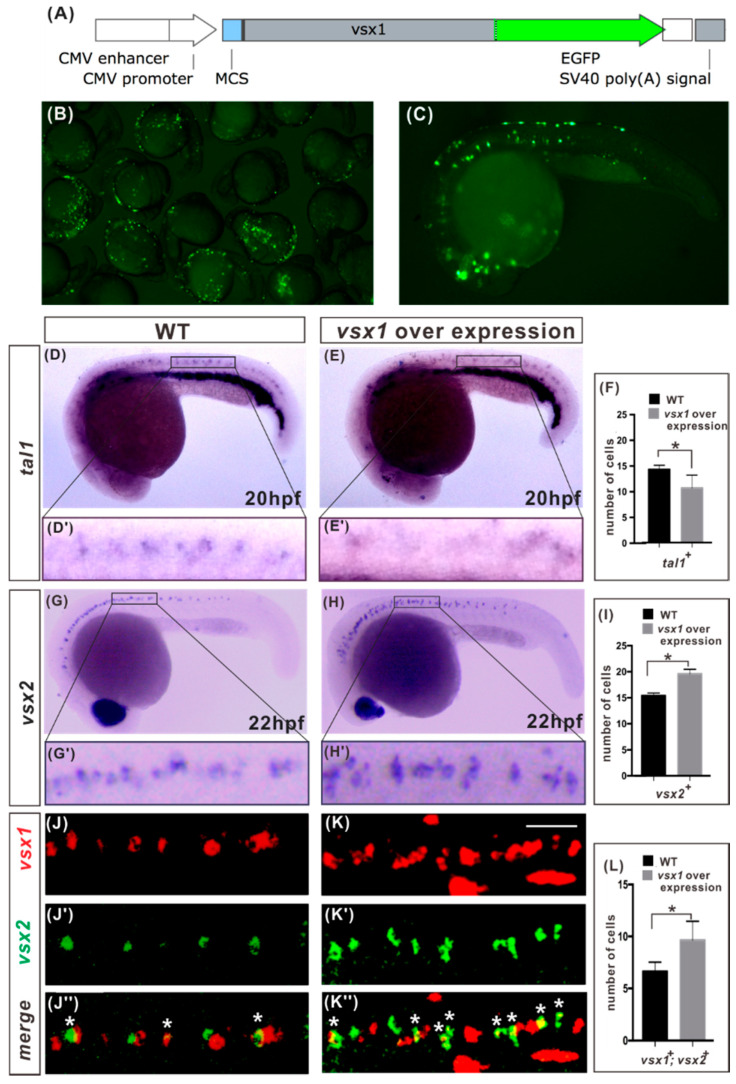
Vsx1 overexpression results in repression of *tal1* and expansion of *vsx2* expression. (**A**) Schematic diagram of *vsx1–egfp* fusion gene expression plasmid. (**B**) Screened embryos with *vsx1*–*egfp* expression in the spinal cords. (**C**) Magnified lateral view of an embryo in image B. (**D**,**E**,**G**,**H**) Whole-mount in situ hybridization of *tal1* and *vsx2* expression in wild-type and *vsx1* overexpression embryos. (**F**,**I**) Quantification of marked neurons number show that the number of *tal1*^+^ cells decreased by 33%, while the number of *vsx2*^+^ cells increased 32% in *vsx1* overexpression embryos. (**J**,**K**) Fluorescent double in situ hybridization of *vsx1* (red) and *vsx2* (green) expression in wild-type and *vsx1* overexpression embryos. (**L**) Quantification of marked neurons number showed that *vsx1* and *vsx2* co-expressing cells increased by 30% in *vsx1* overexpression spinal cords at 22 hpf, which is well consistent with the increased proportion of *vsx2*^+^ cells shown in I. The injected reagents are indicated at the tops of images and makers are indicated on the left sides of images. Dorsal is upwards; anterior is leftwards. Scale bars: 25 μm. Asterisks indicate double-labelled cells. Counts of *vsx1–vsx2* co-expressing cells were derived from both sides of spinal cord above the yolk extension over a five-somite distance. Data in wild-type (black) and overexpression (gray) are presented as means ± SEMs in F, I and L from 10 embryos from at least two independent experiments. Statistical significance was assessed using the unpaired two-tailed Student’s *t*-test. * *p* ≤ 0.05.

To directly investigate if Vsx1 can repress *tal1*, we examined *tal1* expression in the spinal cords of *vsx1*-overexpressing embryos by WISH. Because injection of *vsx1* mRNA can severely inhibit notochord and spinal cord formation [23], we constructed a *vsx1* expression plasmid in which *vsx1* was linked with an enhanced green fluorescent protein (*egfp*) reporter gene (Figure 3A). When 320pg of *vsx1–egfp* fusion gene expression plasmid was injected at one cell stage, its expression at cleavage and gastrula stages was very weak, and thereby availably alleviated the deformity effect of Vsx1 overexpression on the embryos. Given the mosaicism of the expression of the vsx1–egfp fusion, *vsx1*-overexpressing embryos were screened in advance by the expression of *egfp* under fluorescent microscope at 20 hpf and 22 hp; only those embryos with normal dorsal midline structures and detectable expression of *vsx1–egfp* in the spinal cord were selected for further analysis (Figure 3B,C). WISH analysis showed that the number of *tal1*-expressing V2b cells was decreased by 33% in the spinal cord V2 domains of examined *vsx1* overexpression embryos at 20 hpf (Figure 3D–F). At the same time, the number of *vsx2*-expressing V2a cells was increased about 32% in the spinal cords of examined *vsx1* overexpression embryos at 22 hpf (Figure 3G–I). These observations revealed that *vsx1* overexpression can repress *tal1* transcription and result in ectopic *vsx2* expression in the spinal cord V2 domain. To validate that this is a cell autonomous effect on *vsx2* expression, we carried out fluorescent WISH of *vsx1* and *vsx2* and observed that there were more *vsx1–vsx2* co-expressing cells in *vsx1* overexpression embryos than in the wild type embryos at 22 hpf (Figure 3K,L). These results demonstrate that *vsx1* ectopic expression in the spinal cord V2 domain can elicit ectopic *vsx2* expression and V2a generation in a cell autonomous manner.

### 2.4. Vsx1 Can Directly Repress tal1 Transcription

**Figure 4:**
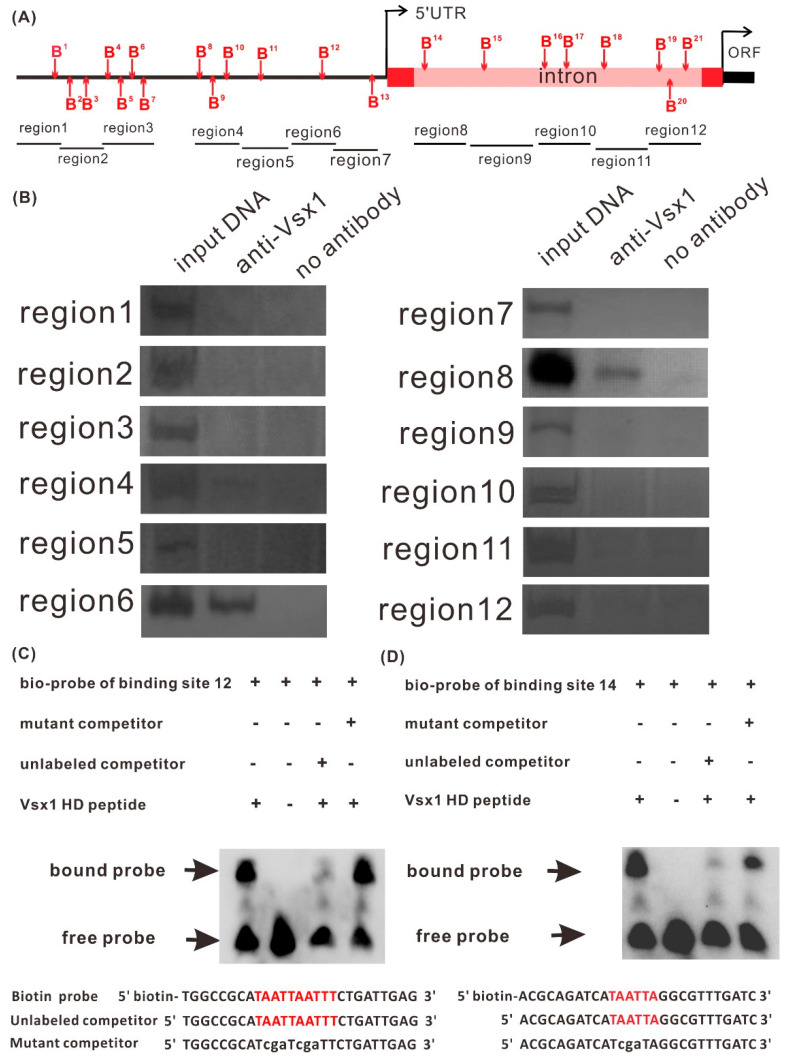
Vsx1 directly binds to *tal1* proximal promoter at two specific sites. (**A**) The positions of potential Vsx1 binding sites B1–B21 at the proximal promoter of *tal1*. The labels region 1–18 represent the examined regions in ChIP assay. (**B**) ChIP assay on extracts from wild type embryos. Input is the positive control with the sonicated original genomic DNA fragment. No antibody immunoprecipitation was used as negative controls for ChIP assay specificity. These results show that *tal1* promoter region 6 and region 8 can recruit Vsx1. (**C**–**D**) Gel electrophoretogram of EMSA of potential binding sites 12 and 14 in region 6 and 8, respectively. The sequences of biotin *tal1* probe and unlabeled wild-type and mutant competitors are shown at the bottom. Red and lower cases indicate the wild-type and mutant bases respectively, at binding sites 12 and 14.

It has been proven that Vsx1 can bind to TAATTN sequence in the promoter of target gene and directly repress its expression [23]. Sequence analysis revealed that there are 21 potential Vsx1 binding sites distributed in the 4 kb long proximal promoter region upstream of the *tal1* transcription start position and in the 2kb long intron located in the 5’ UTR (Figure 4A, Figure S7). To determine Vsx1 directly or indirectly represses *tal1*, we first examined whether Vsx1 could bind to the potential binding sites by chromatin immunoprecipitation (ChIP) assay in wild-type embryos at 24 hpf. After immunoprecipitation with the anti-Vsx1 antibody, the binding of Vsx1 to all the 21 potential binding sites was examined by specific PCR with 12 pairs of primers (Figure 4A) using the genome fragments from immunoprecipitation as the templates. PCR products were always amplified with the primer pairs spanning the potential binding site 12 in the region 6 and binding site 14 in the region 8 (Figure 4B). Sequence analysis confirmed that the PCR products were identical to that of the corresponding *tal1* promoter region. No PCR products were detected in the other regions (Figure 4B). These results indicate that Vsx1 can bind to *tal1* proximal promoter at sites 12 and 14.

Electrophoretic mobility shift assay (EMSA) was performed to examine whether Vsx1 homeodomain directly binds to the two binding sites. After fusion peptide Vsx1–Homeodomain–His was incubated with the two biotin-5′end-labeled probe designed based on the binding site 12 and 14 sequences, the binding complexes were detected with a much slower motility (Figure 4C,D). When 500-fold of excess unlabeled competitor probe was pre-incubated before incubation with biotin-labeled probe, only a weak band of binding complex was detected (Figure 4C,D). By contrast, pre-incubation with 500-fold or more of excess unlabeled mutant probes, in which the TAATTA was converted into TCGATA, exhibited no competitive effect (Figure 4C,D). These results proved that Vsx1 can directly bind to the binding sites 12 and 14 at *tal1* promoter.

Similar to the Vsx1 binding sites in the promoters of *flh* and *ntl* genes [23,24], both actual binding sites, 12 and 14, are located in the GC rich region (Figure S7). These data suggest that the core consensus sequence TAATTN for homeodomain binding is essential but insufficient in determining Vsx1 binding site, and the GC-rich region might contain cis-elements or form a specific spatial structure for Vsx1 to recognize the binding site in vivo.

### 2.5. Both Binding Sites, 12 and 14, Play a Role in Mediating the Repression of tal1 Transcription by Vsx1

**Figure 5:**
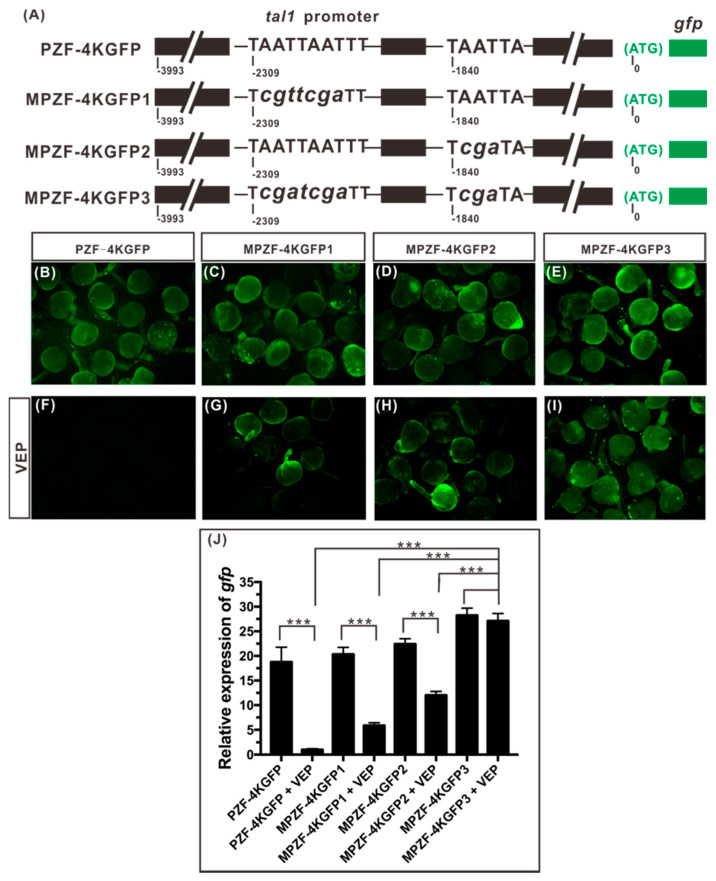
Both binding sites, 12 and 14, in *tal1* proximal promoter are essential for Vsx1 to inhibit *tal1* expression. (**A**) Diagram of GFP report sensors driven by wild-type or different mutant *tal1* proximal promoter. Lower cases indicate the mutant bases at the binding sites 12 and 14. (**B**–**I**) VEP: *vsx1* expression plasmid. GFP expression driven by wild-type or different mutant *tal1* proximal promoter at 24 hpf. The types of GFP reporter sensors are shown at the tops of the images, and the co-injected *vsx1* expression plasmid is shown on the left of the images. (**J**) Quantitative analysis of Vsx1-dependent *tal1* repression mediated by different mutant *tal1* proximal promoters. Results are expressed as means ± SEMs, and statistical analyses were done by unpaired *t*-test. *** indicating statistically significant difference (*p* < 0.001).

To verify that Vsx1 can repress *tal1* expression from the two TAATTN motifs at binding sites 12 and 14, we examined whether full-length Vsx1 protein can inhibit the expression of GFP reporter gene driven by wild-type or mutant *tal1* proximal promoter. Four GFP reporter gene sensors driven by a 4kb long wild-type or different mutant *tal1* proximal promoter were constructed (Figure 5A). The 4kb long *tal1* proximal promoter in PZF-4KGFP was the wild type. In MPZF-4KGFP 1, the TAATTAATTT motif at binding site 12 was converted to TCGATCGATT. In MPZF-4KGFP 2, the TAATTA motif at binding site 14 was converted to TCGATA. In MPZF-4KGFP 3, both binding sites, 12 and 14, were the converted mutant types as described in MPZF-4KGFP 1 and 2.

Detection of green fluorescence under a fluorescence microscope showed that the wild type and the three mutant *tal1* proximal promoter fragments did not initiate GFP expression before the tail bud stage (data not shown) but drove GFP expression successfully and ubiquitously during somite formation stages (Figure 5B–E). Therefore, we co-injected *vsx1* expression plasmid (encoding full-length Vsx1 protein) rather than *vsx1* mRNA with the GFP reporter sensors at one-cell stage to examine the effect of Vsx1 on repressing *egfp* expression from binding sites 12 and 14. When 138 pg *vsx1* expression plasmids was co-injected separately with 390 pg each of the four GFP reporter gene sensors, the expression of GFP was successfully suppressed from the wild-type PZF-4KGFP (Figure 5F) but was not from the three mutant MPZF-4KGFPs (Figure 5G–I). This result substantiated that Vsx1 protein can specifically bind to sites 12 and 14 within *tal1* promoter and play a role in repressing the downstream gene expression.

Real-time quantitative RT-PCR analysis further revealed that, compared with the wild type PZF-4KGFP injected controls, the transcriptional level of *egfp* was significantly increased in each of the different mutant MPZF-4KGFP injected embryos. However, the transcription of *egfp* from the mutant MPZF-4KGFP1 and 2 was still partially suppressed; only that from the double mutant MPZF-4KGFP 3 was no longer suppressed (Figure 5J). The transcription of *egfp* in double mutant MPZF-4KGFP 3 along injected embryos and co-injected with *vsx1* expression plasmids embryos was at the same high level. These results indicate that both binding sites 12 and 14 play a role in mediating the repression of *tal1* transcription by Vsx1.

## 3. Discussion

In zebrafish, Vsx1 is initially expressed in the common P2 progenitors of V2a and V2b interneurons. With the division of pair-producing progenitors, Vsx1 expression was diminished in tal1-expressing V2b cells, while being transiently maintained in presumptive V2a cells. [11,12]. In this study, we investigated whether Vsx1 plays a role in V2a and V2b interneuron fate diversification in this species. When the function of zygotic Vsx1 was blocked, the number of *tal1*-expressing V2b interneurons was increased while the number of excitatory V2a interneurons was decreased in an almost equal proportion in the spinal cord. By contrast, in *vsx1* overexpression embryos, the normal *tal1* transcription in the presumptive V2b cells was repressed, while *vsx2*-expressing V2a cells were ectopically generated. Chromatin immunoprecipitation and electrophoretic mobility shift assays in combination with core consensus sequence mutation analysis further revealed that zygotic Vsx1 can directly repress *tal1* by binding to *tal1* promoter at two sites. Taken together, our results demonstrated that zygotic Vsx1 is essential for preventing *tal1* ectopic expression in presumptive V2a cells and preserving normal V2a interneuron fate specification during V2a and V2b sub-lineage diversification in zebrafish.

### 3.1. Vsx1 Plays a Role in Stimulating vsx2 Expression in V2a Interneuron Sub-Lineage in Zebrafish

During the diversification of V2a and V2b cells, *vsx2* is steadily expressed in V2a but not in *tal1*-expressing V2b cells in all the examined vertebrates [11,22]. In zebrafish embryonic spinal cords, the expression of *vsx1* initiated in the common P2 progenitors is transiently maintained in the V2a sub-lineage, and a significant number of V2a cells co-express *vsx1* and *vsx2* in wild-type embryos [11], suggesting that *vsx2* and *vsx1* are expressed in the same cells but in a temporally distinct manner.

In different tissues, the effect of Vsx1 on the expression of its paralog Vsx2 seems to be different. It has been observed that Vsx1 can inhibit *vsx2* in the type 7 cone bipolar cells of mice [29]. However, Vsx2 distribution is not expanded in the absence of Vsx1 in mouse spinal cord [22]. In zebrafish Notch signaling mutants, the numbers of both *vsx2*-expressing cells and *vsx1*-expressing cells are increased [11]. In this study, we observed that *vsx2* expression was significantly inhibited in Vsx1 knockdown embryos and expanded in Vsx1 overexpression embryos. These observations suggest that Vsx1 plays a role in stimulating *vsx2* expression in V2a interneuron sub-lineage during the development of spinal cord in zebrafish.

In vertebrate spinal cord, Lhx3 is expressed in both P2 progenitors and motor neuron progenitors. It has been observed that Lhx3 is able to promote Vsx2 expression in the developing spinal cord [17,18]. However, in normal development of spinal cord, Vsx2 is expressed only in the progeny cells of P2 progenitors that co-express Vsx1 and Lhx3 [9,10,11,22] but not in the progeny cells of motor neuron progenitors in which *lhx3* is not expressed with *vsx1* [11,22]. These phenomena suggest that Lhx3 alone is unable to promote Vsx2 expression. Moreover, the number of *lhx3*-expression cells and the generation of motor neurons were not affected in zygotic *vsx1* knockdown and transient directed gene knockout embryos (Figure 1M and Figure S4F), implying that zygotic Vsx1 has no impact on the expression of Lhx3 in both motor neurons and V2a interneurons. It is possible that that Vsx2 expression program is triggered by the co-operation of Vsx1 and Lhx3 in P2 progenitor cells.

### 3.2. Direct Repression of tal1 by Paired-Like Homeodomain Repressor might Be an Evolutionarily Conserved Mechanism of V2a and V2b Diversification

During V2 interneuron development, Vsx1 and Vsx2 are expressed in the V2 precursors and mature V2a cells, respectively [11,12,19,20,21,22]. Like Vsx1, Vsx2 contains an evolutionarily conserved homeodomain and a CVC domain [30,31]. The homeodomain can directly band to TAATTA motif [32,33] and the CVC domain plays an essential role in assisting the homeodomain in high-affinity DNA binding [34,35]. This similarity implies that Vsx2 also has the ability of binding to *tal1* promoter and repressing *tal1* transcription. Since Vsx1 is only transiently maintained in V2a sub-lineage at early specification stage, the subsequent steady expression of Vsx2 may ensure the persistent inhibition of Tal1 erroneous expression in V2a interneuron sub-lineage. A recent study in mice has observed that Vsx1 and Vsx2 act successively to secure V2 interneuron identity and Vsx2 can inhibit Gata3 expression and V2b interneuron differentiation [36]. We have reason to believe that, in zebrafish, the transient maintenance of Vsx1 in V2a cells inhibits Tal1 expression and stimulates Vsx2 expression at the onset of V2a and V2b diversification, resulting in adequate segregation of Vsx2 and Tal1 expression and proper V2a vs. V2b fate decision.

Unlike in zebrafish, the expression of Vsx1 is detected in common V2 precursors but not maintained in V2a sub-lineage at the onset of V2a and V2b diversification, and Vsx1 exhibits no detectable impact on proper generation of V2 interneuron subsets in the mouse’s embryonic spinal cord [22]. These observations suggest that Vsx1 might be not involved in the inhibition of Tal1 in this species. However, the expression program of Tal1 is triggered in the common V2 precursors by the Foxn4-drivn regulatory network, and erroneous expression of Foxn4 and its target genes can elicit ectopic expression of Tal1 and ectopic formation of V2a interneurons in the developing mouse spinal cord [7,14]. Therefore, repressing the erroneous expression of *tal1* in V2a cells is also an essential mechanism for normal V2a interneuron sub-lineage specification in mice. It is possible that, in mice, the expression of Tal1 in V2a interneuron sub-lineage is inhibited by Vsx2 alone and direct repression of *tal1* transcription by paired-like homeodomain regulator Vsx1 or Vsx2 might be an evolutionarily conserved mechanism of V2a and V2b diversification.

### 3.3. Notch-Delta Signaling may Activate Ubiquitin-Dependent Proteolysis of Vsx1 to Promote tal1 Transcription

Since Vsx1 is expressed in the common P2 progenitors of V2a and V2b cells in zebrafish [11,12,19], its products will be inherited to the V2 daughter cells after cell division. Indeed, Vsx1 is transiently maintained in both the newly generated V2a and V2b cells [11,12]. Given that Vsx1 can repress *tal1* expression, a downregulation of Vsx1 is required for the expression of *tal1* in presumptive V2b cells at the onset of V2 cells diversification in zebrafish. How Vsx1 expression remains in V2a cells but is repressed in V2b cells in this species remains, however, an open question that would be interesting to address.

It is well known that Notch signaling activates *tal1* expression to promote V2b interneurons differentiation [10,11]. In both zebrafish and mice, *mib* encodes an E3 ubiquitin ligase that is essential for efficient activation of Notch signaling [37,38] and Vsx1 is known to be a substrate of the ubiquitin/proteasome pathway [39]. In zebrafish *mib* mutants, which lack Notch signaling due to the blocking of ubiquitination process, there is a dramatic expansion of *vsx1* expression and concomitant with the loss of *tal1* expression in the spinal cord [10,11]. By contrast, forced activation of Notch signaling in zebrafish embryos resulted in loss of *vsx1* expression in paired P2 sibling cells [12] and concomitant with a vast increase of *tal1* transcripts [10]. These observations suggest that, in zebrafish, Notch-delta signaling might repress Vsx1 expression, either directly or indirectly, to protect *tal1* transcription and promote V2b differentiation.

## 4. Materials and Methods

### 4.1. Zebrafish Maintance and Embryo Acquisition

Zebrafish were maintained under standard conditions at 28.5 °C in a 14 h/10 h light/dark cycle with approval by the Institutional Animal Care and Use Committee at Zhejiang University (Zju 201306-1-11-060). Embryos were obtained from natural spawning and staged by hours post-fertilization (hpf) at 28.5 °C as well as by morphology, as described previously [40].

### 4.2. Morpholino Oligonucleotides

*vsx1* splice-blocking Morpholino antisense oligonucleotide (sbMO) was synthesized by Gene-tools (Philomath, OR). The sequence of *vsx1* sbMO is 5’-AGCAAAGTGATTCGTACCGGAGTAA-3’, the same as the published *vsx1* sbMO sequence [25].

### 4.3. Transient Knockout of vsx1 by CRISPR/Cas9

The sgRNA targeting to exon 1 of *vsx1* was designed on a website (http://chopchop.cbu.uib.no/). The template for sgRNA transcription was amplified by PCR with Q5 polymerase (M0492LL, NEB, Boston, MA, USA) and a pair of primers as described in a published paper [41]. The sequences of the primers were 5’-GAAATTAATACGACTCACTATAGGATTTGTCGATTCCGAACGAGTTTTAGAGCTAGAA-3’ (forward) and 5’-AAAAGCACCGACTCGGTGCCACTTTTTCAAGTTGATAACGGACTAG CCTTATTTTAACTTGCTATTTCTAGCTCTAAAAC-3’ (reverse, underlined letters indicate the target sequence). sgRNA was transcribed in vitro with T7 transcription kit (AM1334, Invitrogen, Carlsbad, CA, USA) and purified by MEGAclear kit (AM1908, Invitrogen, Carlsbad, CA, USA). Cas9 protein (A36498, Invitrogen, Carlsbad, CA, USA) was diluted to 0.6 μg/μL and sgRNA diluted to 0.3 μg/μL. Then a mixture of Cas9 protein (0.3μg/μL) and sgRNA (0.15μg/μL) was made and incubated for 5 min at 37 °C. Directed gene knockout for screening in G0 zebrafish was done by injecting the mixture of sgRNA and Cas9 at one cell stage as described by Wu et al. [42].

Two days after microinjection, each selected embryo was lysed by 30 μL of 40 mM NaOH buffer at 95 °C for 30 min; then 3 μL Tris-HCL (pH 6.0) was added and mixed. A 295 bp DNA fragment contain the target site was amplified from the lysed product by PCR with a pair of primers (forward primer: 5’-CTATACAGCAAGAGTGACCTC-3’, Reverse primer: 5’-AAGTCTCTGTTTCCTCCGCAG -3’) and sequenced.

### 4.4. Construction of vsx1–egfp Fusion Gene Expression Plasmids

*vsx1–egfp* fusion gene expression plasmid was constructed by inserting *vsx1* CDS into the pEGFP-1 plasmid (BD bioscience, San Jose, CA, USA) at the position immediately upstream of the coding region of *egfp* gene. *vsx1* CDS without termination codon was amplified by PCR with primers 5’- CAGGACGAATTCATGACGGGAAGAGAAGAAGCT-3’ (forward) and 5’-GGGC GCTCGAGACTCTCATTTTCAGAATCGCTG-3’ (reverse, the restriction endonuclease recognition sites EcoRI and XhoI are underlined). The primers used in amplifying *vsx1* CDS were designed according to the sequence of zebrafish *vsx1* (GenBank accession number: NM_131333.1). The recombinant *vsx1–egfp* fusion gene without frameshift mutations was confirmed by sequencing.

### 4.5. Cloning of Proximal Promoter Sequence of Zebrafish tal1 and Construction of GFP Sensors

A 4 kb *tal1* promoter fragment upstream of the ATG was amplified from zebrafish genomic DNA using primers: 5’-AGAGCTCGATGGAGCTGATTTCCCAC-3’ (forward) and 5’-TTATGGATCCCCTTCGGCGCGATCCCAGAA-3’ (reverse, the restriction enzyme sites SacI and BamHI are underlined), which were designed according to the sequence of zebrafish *tal1* (GenBank accession number: NC_007133.7). This fragment was then inserted into SacI and BamHI sites of vector pEGFP-1 (Clontech, Mountain View, CA, USA) with the enchanced GFP reporter gene to construct PZN-4KGFP plasmid. Mutant MPZN-4KGFP plasmids were generated by inducing the mutant sequence at the desired site with Mut Express^®^ II Fast Mutagenesis Kit (Vazyme, Nanjing, China) and different specific primer pairs. The primer pair used in generating mutant MPZN-4KGFP1 was 5’-GCCGCATcgatcgaTTCTGATTGAGCCCTGAAGCAG-3’ (forward) and 5’-TCAGAAtcgatcgATGCGGCCAAAAGTAACACA TTTTCAG-3’ (reverse, lower cases indicate the mutant sites). The primer pair used in generating mutant MPZN-4KGFP2 was 5’-GCAGATCATcgaTAGGCGTTTGATCGACGCGAGAT-3’ (forward) and 5’-AACGCCTAtcgATGATCTGCGTTAGTATCAAAATAGCGC-3’ (reverse, lower cases indicate the mutant sites). The primer pairs above-mentioned were all used in generating mutant MPZN-4KGFP3. All recombinants were reconfirmed by sequencing and extracted by Endo-Free Plasmid Mini Kit (Omega Biotech Corporation, Norcross, GA, USA).

### 4.6. Microinjection

All the samples were injected into the blastodisc at the 1 to 2-cell stage. For co-injection, the desired samples were mixed thoroughly prior to injection.

### 4.7. Western Blotting

Embryos were frozen in liquid nitrogen and lysed in Tris-SDS buffer (60 mM Tris-HCL, PH6.8, 3.5% SDS). The lysate of each embryos was centrifuged for 7 min at 500g at 4 °C; then we added 2μL Tris-SDS buffer to pellet. Mixtures were heated for 10 min at 100 °C and centrifuged for 5 min at 14,000 rpm at 4 °C. Supernatant was separated by electrophoresis on 8% SDS-PAGE and then the samples were transferred to PVDF membranes (F619537, Sangon, Shanghai, China). Membranes were blocked for 1 h in TBST with 5% non-fat milk powder. The rabbit polyclonal antibody anti-zebrafish Vsx1 was made by Jiaxuan corporation using Vsx1 N terminal 1-144 amino acid as antigen. The Vsx1 primary antibody was diluted 500 times and the HistoneH3 antibody (A2348, ABclonal, Wuhan, China) was diluted 1000 times with 5% milk/TBST solution before used. Membrane was incubated with the diluted Vsx1 antibody and HistoneH3 antibody separately at 4 °C overnight. After being washed for 4 × 10 min with TBST, the member was incubated with secondary antibody (AS014, ABclonal, Wuhan, China) for 1 h at room temperature. Membrane was washed for 4 ×10 min with TBST and stained with ECL kit (36208ES60, Yeasen, Shanghai, China). Band intensity was quantified in ImageJ software and Vsx1 protein was normalized to a corresponding Histone H3 value.

### 4.8. Digoxigenin or Fluorescein-Labeled RNA Probe Synthesis

The full or partial lengths of cDNAs for *tal1* (NM_213237.1), *vsx2* (NM_131462), *eng1b* (NM_001013498), *islet1* (BC060892), *islet2* (NM_130970), *foxn4* (NM_131099.2), *slc17a6b* (AB183386.1), *gad1b* (AB183390.1), *sox1a* (AB242327.1), *sox1b* (AB242328.1) and *vsx1* (NM_131333.1) were obtained by RT-PCR. The used primers are shown in Supplementary Table S1. The fragments were separately inserted into the pBluescriptII SK (+/−) vector (Stratagene, Bellinghan, WA, USA). Then Digoxigenin or Fluorescein-labeled antisense RNA probes were transcribed with T7 or T3 RNA polymerase, respectively, in the presence of Digoxigenin or Fluorescein mix (Roche, Switzerland) from the linearized plasmids. The newly synthesized RNA probes were then purified by Quick Spin Columns (Roche, Basel, Switzerland) and stored at −80 °C.

### 4.9. Whole Mount In Situ Hybridization (WISH)

Whole mount in situ hybridization was carried out as described in published paper [43]. Imaging was performed using a Nikon MODEL C-DSS230 (DIC images). Whole mount fluorescent in situ hybridization using enhanced tyramide signal amplification was carried out as described [44]. For confocal imaging, embryos were mounted at 20 hpf in 1% low-melting point agarose. Imaging was performed using a confocal microscope (LSM 710, Carl Zeiss, Jena, Germany) with a 25× water immersion objective.

### 4.10. Cell Counts and Statistical Analyses

In all cases, cell counts were carried out as described [16], from both side of a 5 or 7-somite length of spinal cord adjacent to somites 6–10 or somites 6–12. For each experiment, details are presented in figure legends. Normal distribution and homogeneity of variance were analyzed by K–S test or F test, respectively, in advance, to decide which of the statistical analysis methods—Wilcoxon–Mann–Whitney test, unpaired Student *t* test or unpaired Student *t* test with Welch’s correction, was to be used for evaluating statistical significance.

### 4.11. Chromatin Immuno Precipitation (ChIP)

ChIP experiments were performed using the ChIP-IT Express kit (Activemotif, California). Wild type embryos at 24 hpf were fixed in 1% formaldehyde for 10 min, after incubation in ice-cold lysis buffer with 5 µL PIC and 5μL PMSF for 30 min, grinded them on ice with Dounce homogenizer to aid in nuclei release. We centrifuged the embryos for 10 min at 5000 rpm in 4 °C, removed the supernatant and resuspended the nuclei in shearing buffer. It was then sonicated and produced genome fragments between 200–400 bp. After sonication, 10 μL of supernatant was collected as input DNA; 60 μL (25 μg) of supernatant was incubated overnight at 4 °C with protein G magnetic beads and rabbit polyclonal antibody against Vsx1 [23]. After washing beads and reverse cross-links, the DNA sample was then incubating at 95 °C for 15 min and digested with proteinase K, then it could be used in PCR analysis. Primers used in amplifying different regions of *tal1* proximal promoter containing potential Vsx1 binding sites were designed according to the zebrafish *tal1* genomic sequence (GenBank accession number: NC_007133.7). The sequences of primer pairs are shown in Supplementary Table S2.

### 4.12. Electrophoretic Mobility Shift Assay (EMSA)

Vsx1 homeodomain (residues 132–224) were expressed with prokaryotic expression vector pGEX-4T-1 as in He et al. [23]. Oligonucleotides were 5′ end labeled with biotin. The sequences of biotin labeled probe, unlabeled wild type and mutant competitive probes are indicated in Figure 4C and D. EMSA was performed using LightShift Chemiluminescent EMSA Kit (Pierce, Rockford, IL, USA). Binding reaction and protein: DNA mixes separation were performed as described previously [23]. After electrophoresis on 6% non-denaturing polyacrylamide gels, oligonucleotides were transferred onto nylon membrane through electro blotting. Biotin-labeled probes were detected by Chemiluminescent Nucleic Acid Detection Module (Thermo, Waltham, MA, USA), exposure and photographed by BIO-RAD ChemiDoc^TM^ Touch Imaging System. In competing experiments, different unlabeled probes were incubated with the purified Vsx1 Homeodomain peptide ahead the incubation with labeled probe.

### 4.13. Quantitative RT-PCR

Real-time quantitative polymerase chain reaction (RT-qPCR) was performed in a Light-Cycler^®^ 480 System (Roche, Germany). *ef1α2* was employed as the internal standard. Total RNA was extracted from the EGFP reporter injected zebrafish embryos at 24 hpf using TRIzol reagent (Ambion, Austin, TX, USA). Full length cDNA was obtained through reverse transcription using SYBRs Prime-ScriptTM RT-PCR Kit (TaKaRa, Osaka, Japan) according to the manufacturer’s instructions. RT-PCR primers sequences were as follows: 5′-CTGCTGCCCGACAACCA-3′ (*egfp* forward), 5′-TGTGATCGCGCTTCTCGTT-3′ (*egfp* reverse), 5′-CCAACTTCAACGCTCAGGTCA-3′ (ef1α2 forward), 5′-CAAACTTGCAGGCGATGTGA-3′ (ef1α2 reverse). For each sample, the test and control reactions were run in triplicate. After the reaction, we used the threshold cycle (Ct) values of 2-ΔΔCT to calculate the relative expression of *egfp* in different samples by qRT software provided for the Light Cycler^®^ 480 System. The histogram for fold comparison of different samples and unpaired *t* test was generated by the GraphPad Prism7 program software.

## 5. Conclusions

*vsx1* knockdown and transient knockout leads to ectopic expression of *tal1* in the presumptive V2a cells of the zebrafish spinal cord and suppression of excitatory V2a interneuron generation. By contrast, *vsx1* overexpression suppresses normal *tal1* transcription in the presumptive V2b cells and elicits ectopic generation of V2a interneurons in the spinal cord. Regulatory mechanism analysis shows that Vsx1 can directly repress *tal1* by binding to *tal1* promoter at two specific sites. These results indicate that Vsx1 plays an essential role in V2a fate specification during V2a and V2b sub-lineage diversification by directly preventing *tal1* expression in presumptive V2a cells.

## Supplementary Materials

The following are available online at https://www.mdpi.com/1422-0067/21/10/3600/s1. Figure S1: CRISPR/Cas9 induced vsx1 knockout G0 Mutant. (A) Schemic representation of zebrafish *vsx1* gene. (B) Sequencing spectrums of partial *vsx1* exon 1 region in a wild type and a knockout G0 chimeric mutant embryo. (C) Different mutations detected in a G0 chimeric mutant embryo. Substituted and deleted base are marked in red, Figure S2: Phenotypes of zygotic *vsx1* knockdown embryos at different developmental stage. (A–B) *vsx1* sbMO injected embryos exhibit no detectable morphological and structural deformities in appearance until 27 hpf. (C–D) *vsx1* knockdown embryos showed smaller head and eyes, yet bigger yolk sac than that in the wild type. (C’–D’’) Lateral (C’,D’) and dorsal (C’’,D’’) magnified views of a wild-type and zygotic *vsx1* knockdown embryo. The injected reagents are indicated at the top of images and developmental stage is indicated at the left side of images. Scale bars: 0.4 mm in C’–D’’. (E) Proportion of different phenotypes in wild type and zygotic *vsx1* knockdown embryos at 72 hpf, Figure S3: Phenotypes of chimeric *vsx1* knockout G0 embryos at different developmental stage. (A–B) *vsx1* knockout G0 embryos exhibit no detectable morphological and structural deformities in appearance as vsx1 sbMO injected embryos until 27 hpf. (C–D) Comparison of wild type and *vsx1* chimeric knockout Go embryos at 72 hpf. (E) Lateral views of wild type and *vsx1* chimeric knockout G0 embryos. *vsx1* knockout G0 embryos at 72 hpf exhibit small head and small eyes deformities as observed in *vsx1* knockdown embryos at 72 hpf. The injected reagents are indicated at the top of images. Scale bars: 0.5 mm in E, 1.5 mm in A,B. (F) Proportion of different phenotypes in wild type and *vsx1* knockout G0 embryos at 72 hpf, Figure S4: Occurrence of V1 interneurons and motor neurons are normal in *vsx1* knockdown embryos at 20 hpf. (A–B) V1 interneurons marked by the expression of *eng1b* in wild-type and *vsx1* knockdown embryos. (C–D) Motor neurons marked by the expression of *isl1* and *isl2* (probes mixed) in wild-type and *vsx1* knockdown embryos. The injected reagents are indicated at the top of images and maker is indicated at the left side of images. Dorsal is upwards; anterior is leftwards. Scale bars: 25 μm. (E,F) Quantitative analysis of V1 (E) and motor neuron (F) cells in wild type and *vsx1* knockdown embryos. Counts of *eng1b*-, *isl* & *2*-expressing cells are derived from both sides of spinal cord above the yolk extension over a seven-somite distance. Data in wild-type (black) and *vsx1* knockdown (gray) are presented as mean±s.e.m. in E, F from 10 embryos from at least two independent experiments. Statistical significance was assessed using the unpaired two-tailed Student’s *t*-test. * *p* ≤ 0.05, ** *p* ≤ 0.01, *** *p* ≤ 0.001, Figure S5: *vsx1* knockdown has no impact on the generation of V2s interneurons at 20 hpf. (A–C) Fluorescent double in situ hybridization of *tal1* (red) and *sox1a* & *sox1b* (probe mixed) (green) in wild type and *vsx1* knockdown embryos at 20 hpf. White dotted lines indicate the boundary between adjacent neuron regions. Asterisks indicate double-labelled cells. The injected reagents are indicated at the top of images and maker is indicated at the left side of images. Dorsal is upwards; anterior is leftwards. Scale bars: 25 μm. (C) Quantitative analysis of *tal1^+^-sox1a*&*sox1b^+^* co-expressing cells in wild type and *vsx1* knockdown embryos. Counts of *tal1^+^-sox1a*&*sox1b^+^* co-expressing cells were derived from both sides of spinal cord above the yolk extension over a seven-somite distance from ten embryos. Wild-type (black) and knockdown (gray) values are presented as mean±s.e.m. in C from at least two independent experiments. Statistical significance was assessed using the unpaired two-tailed Student’s *t*-test. * *p* ≤0.05, ** *p* ≤ 0.01, *** *p* ≤ 0.001, Figure S6: Fluorescent double in situ hybridization of *vsx1* (red) and *tal1* (green) shows that *vsx1* and *tal1* are in mutual exclusive expression in wild type embryos at 20 hpf. Marker is indicated at the left side of images. Dorsal is upwards; anterior is leftwards. Scale bars: 25 μm, Figure S7: Potential binding sites of Vsx1 at proximal promoter of tal1 (B1-B21). Yellow region indicates GC-rich region. The putative core element of promoter is in bold. The 5’-UTR and the intron within the 5’-UTR are in red and green, respectively. The start codon is in italics, Supplementary Table S1: Primers used for synthesis of RNA probes.The restriction enzyme sites are underlined, Supplementary Table S2: Primers used for ChIP assay.

## Abbreviations

hpf	hours post fertilization
sbMO	splice-blocking Morpholino
WT	wide type
KD	knock down
CKO	chimeric knock out
WISH	whole mount in situ hybridization
