# Supplementary material for: Zygotic Vsx1 Plays a Key Role in Defining V2a Interneuron Sub-Lineage by Directly Repressing tal1 Transcription in Zebrafish
Source: Int J Mol Sci. 2020 May 20;21(10):3600. doi: 10.3390/ijms21103600 (PMC7279403; doi:10.3390/ijms21103600)
Supplement: Supplementary file 1 [file ijms-21-03600-s001.pdf]

Supporting Information

Zygotic Vsx1 Plays a Key Role in Defining V2a Interneuron Sub-Lineage by Directly Repressing *tal1* Transcription in Zebrafish

Qi Zhang, Haomang Xu, Wei Zhao, Jianbo Zheng, Lei Sun and Chen Luo

Contents:

Supplementary Figures S1-S7.
Supplementary Tables S1-S2

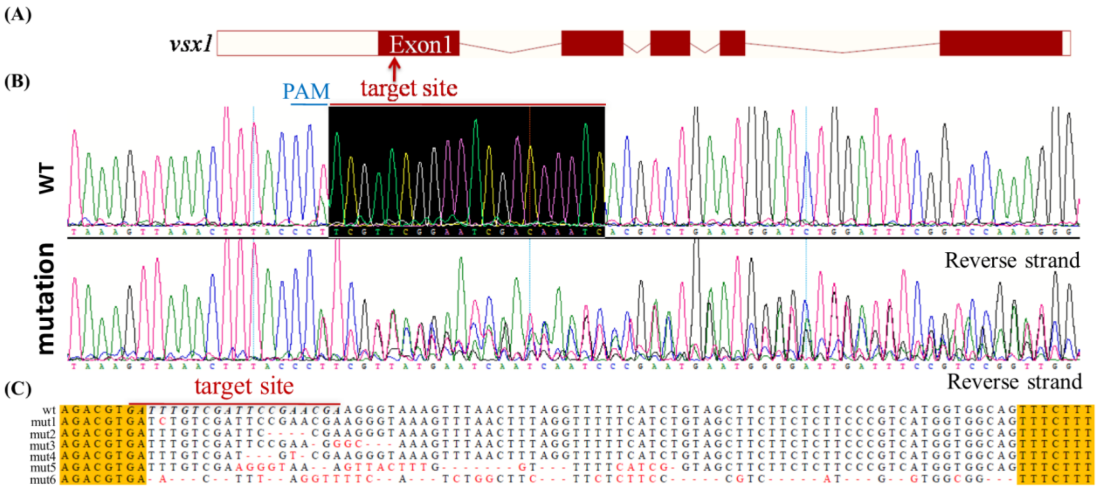

Figure S1. CRISPR/Cas9 induced vsx1 knockout G0 Mutant. (**A**) Schemic representation of zebrafish *vsx1* gene. (**B**) Sequencing spectrums of partial *vsx1* exon 1 region in a wild type and a knockout G0 chimeric mutant embryo. (**C**) Different mutations detected in a G0 chimeric mutant embryo. Substituted and deleted base are marked in red.

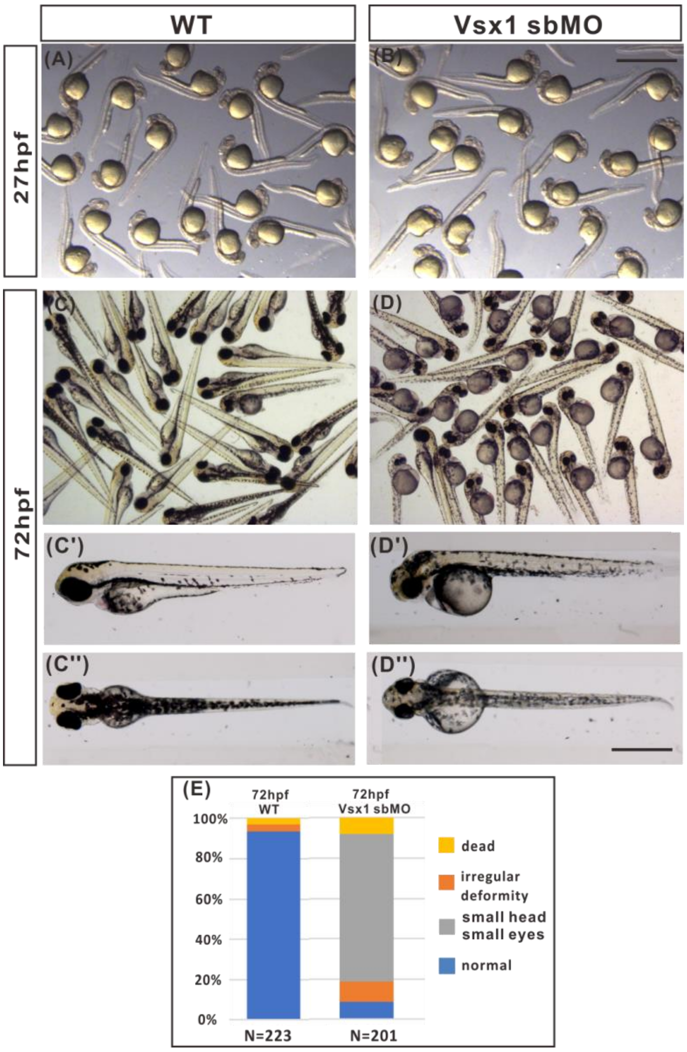

Figure S2. Phenotypes of zygotic *vsx1* knockdown embryos at different developmental stage. (**A–B**) *vsx1* sbMO injected embryos exhibit no detectable morphological and structural deformities in appearance until 27 hpf. (**C–D**) *vsx1* knockdown embryos showed smaller head and eyes, yet bigger yolk sac than that in the wild type. (**C′–D′′**) Lateral (**C′,D′**) and dorsal (**C′′,D′′**) magnified views of a wild-type and zygotic *vsx1* knockdown embryo. The injected reagents are indicated at the top of images and developmental stage is indicated at the left side of images. Scale bars: 0.4mm in C′–D′′. (**E**) Proportion of different phenotypes in wild type and zygotic *vsx1* knockdown embryos at 72 hpf.

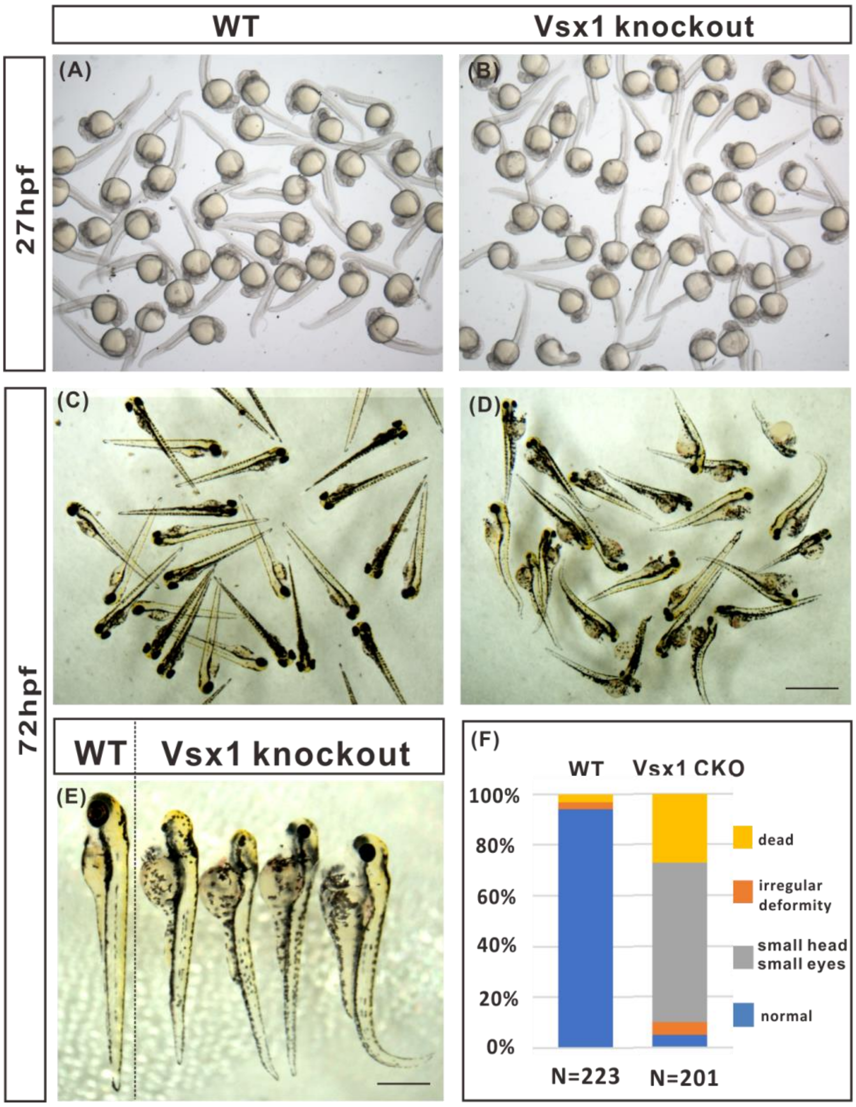

Figure S3. Phenotypes of chimeric *vsx1* knockout G0 embryos at different developmental stage. (**A**–**B**) *vsx1* knockout G0 embryos exhibit no detectable morphological and structural deformities in appearance as vsx1 sbMO injected embryos until 27 hpf. (**C**–**D**) Comparison of wild type and *vsx1* chimeric knockout Go embryos at 72 hpf. (**E**) Lateral views of wild type and *vsx1* chimeric knockout G0 embryos. *vsx1* knockout G0 embryos at 72 hpf exhibit small head and small eyes deformities as observed in *vsx1* knockdown embryos at 72 hpf. The injected reagents are indicated at the top of images. Scale bars: 0.5mm in E, 1.5mm in A,B. (**F**) Proportion of different phenotypes in wild type and *vsx1* knockout G0 embryos at 72 hpf.

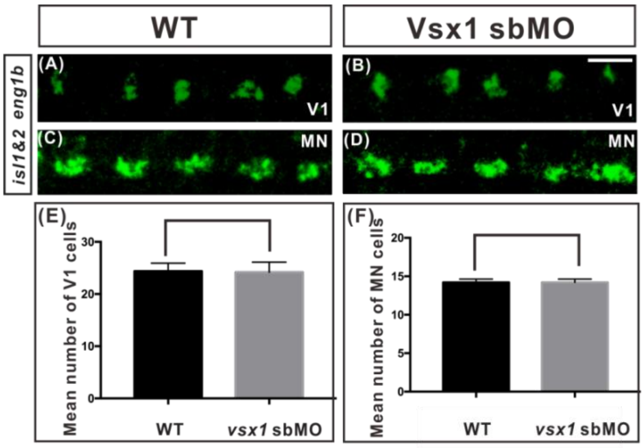

Figure S4. Occurrence of V1 interneurons and motor neurons are normal in *vsx1* knockdown embryos at 20 hpf. (**A–B**) V1 interneurons marked by the expression of *eng1b* in wild-type and *vsx1* knockdown embryos. (**C–D**) Motor neurons marked by the expression of *isl1* and *isl2* (probes mixed) in wild-type and *vsx1* knockdown embryos. The injected reagents are indicated at the top of images and maker is indicated at the left side of images. Dorsal is upwards; anterior is leftwards. Scale bars: 25 μm. (**E,F**) Quantitative analysis of V1 (**E**) and motor neuron (**F**) cells in wild type and *vsx1* knockdown embryos. Counts of *eng1b-*, *isl* & *2*-expressing cells are derived from both sides of spinal cord above the yolk extension over a seven-somite distance. Data in wild-type (black) and *vsx1* knockdown (gray) are presented as mean±s.e.m. in E, F from 10 embryos from at least two independent experiments. Statistical significance was assessed using the unpaired two-tailed Student's t-test. * $p \leq 0.05$, ** $p \leq 0.01$, *** $p \leq 0.001$.

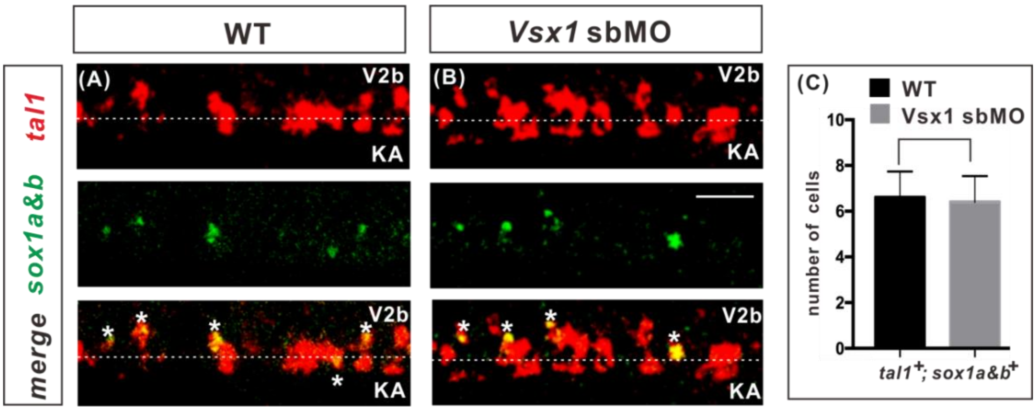

Figure S5. *vsx1* knockdown has no impact on the generation of V2s interneurons at 20 hpf. (**A–C**) Fluorescent double *in situ* hybridization of *tal1* (red) and *sox1a* & *sox1b* (probe mixed) (green) in wild type and *vsx1* knockdown embryos at 20 hpf. White dotted lines indicate the boundary between adjacent neuron regions. Asterisks indicate double-labelled cells. The injected reagents are indicated at the top of images and maker is indicated at the left side of images. Dorsal is upwards; anterior is leftwards. Scale bars: 25 μm. (**C**) Quantitative analysis of*tal1*$^{+}$-*sox1a*&*sox1b*$^{+}$ co-expressing cells in wild type and *vsx1* knockdown embryos. Counts of *tal1*$^{+}$-*sox1a*&*sox1b*$^{+}$ co-expressing cells were derived from both sides of spinal cord above the yolk extension over a seven-somite distance from ten embryos. Wild-type (black) and knockdown (gray) values are presented as mean±s.e.m. in C from at least two independent experiments. Statistical significance was assessed using the unpaired two-tailed Student's t-test. * p ≤0.05, ** p ≤ 0.01, *** p ≤ 0.001.

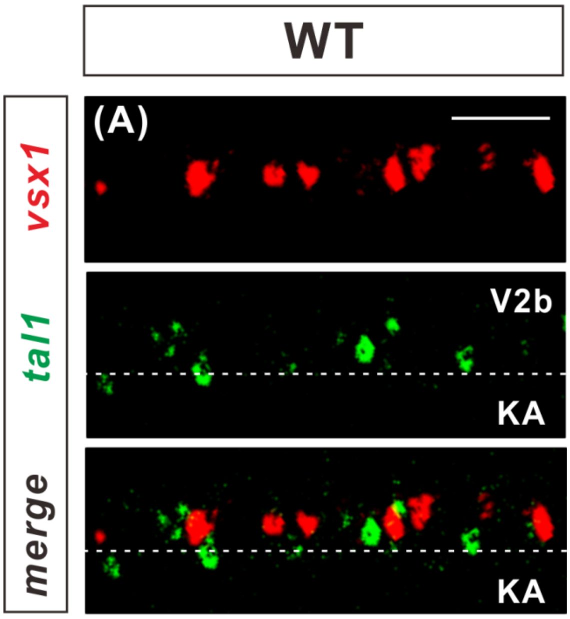

Figure S6. Fluorescent double in situ hybridization of *vsx1* (red) and *tal1* (green) shows that *vsx1* and *tal1* are in mutual exclusive expression in wild type embryos at 20 hpf. Marker is indicated at the left side of images. Dorsal is upwards; anterior is leftwards. Scale bars: 25 μm.

GGGCATCCTTCCCAAATCACTCAAAATCCGTAGCAATGAACTAACAAAGGCCCGAGATCAAAGGCTCT
CAGATGATGGAGCTGATTTCCCACATATTATTCTGCGTGTATTTCATACATTCATTCATGAAGATATATTAT
CACATGTCTGTCTTCTCTATACCTGACACCCCTCTCCGATGTTGACAATTACGGTTATCTGTGTTAATGC
B1

TGGAGTAAATTTGTTGACAACGATCACGGTGATGACGAATTAATGAGCTTCATATCTCCATCATCAGGG
B2

AAACATCTTCAGTAGTCAGTCAACATTTCATTGAAAAGGAAAAACATTTGACGAAATGCATTGCAGCT
TACTATTTTCCCTTGAAATGCTAGCATAGACCATTTTGAGGGATGTAAACAAAAACAATGATCCCATTGT
ATTTTCTTTTTGACATGTTTAATTTCTAAAGCTTCCGAGAATCTAAAAGGAGCCATATTGATTATGATAG
B3

CTGTTTAAACATTGAGTTTTTATTGAATGCCTCTTGTTACAGTTATGAAATAGTTCCATAACAAGCAGGA
AATGTTCATGGGCCAATGACATGATTACATTGGTTTATAGATGGACATTGGGTAACATACAGTTTTGTTT
TATAATTAAATGTTGTGTTTGTCCATATTCTTACCCAACTTTGGGTAAAAAACAACATTTTTAGAGTGTA
B4

TCAATTATGAATTAATTAACTTGAATTTCAAGGATGCACCAGTTATTACATTTTATGACTGTGACCAATAT
B5 B6

ATTAATTAAATAACACTAATATAAAATAGGCAGCAGGGTGGAGCAGTTATAGCACAATCGCCTCACAGC
B7

AAGGAGGTTGAGCCACAGCAGGGTCAGCTGACATTTTTGTGTGGAGTTTGCAATGCATGTTCTCCCCG
TGTTGGTGTGGGCTTCCTCCGGGTGCTCCGGCTTCCCGACAGTCCGAAGTCATGCGGTACAGGTGAAT
TGGATAAGCTAAATTGGCCGTAGTGTATGTGAGTGTGTTCAAGTGTGTGTGGGTGTTTCCCAGTGTTGG
ATTGCGGCTGGAAGGCTATCCGCTGCGTAAAACAGGTGCTGGATAAGTTGGTTCATTCCGCTGTGGCA
ACCCCAAATTAATAAAGTGAATAAGCAAAAAAGAAAATGAACGAATAACTGAATAATATAAATTAAGT
B8 B9

TGAACTAAAGTAAACCATTAGCCTATAAAACATGAAATAGTATATAGAATTTAGTTATTTACAAAATGTT
AAAATTGATCCAAATTATCCTGTATTTTTAGTGCAATAAGGCATTAAAACATTTAAAATGTATAATGTAG
B10

CAAAAACAAAATATTTTAACATGCATGTATGGAGAGCAGGGTGAATAATCTACAATAAAAACTACAAC
ACTGTCCAATTCCGACAAATGTAATAACTGTAAATAATAGAATAAATATTGTGCATCCTGAATAAATACA
TTAGTTTAGTTATCAGGCATTTACAAAAGAAAAAACTCTACCTTAAATTGACATCGAATATCCCCTTAAA
AAATCATTCAGAAAATATTATTCGTGTGCATTCTTGGGTTGGCGTTTGTAATTTGCGTCTGCTTACCTTA
B11

ATAACCTGCCTAACATTTTTACTCACTGAGGGTTATGTTATGAACACGACTTCCAGTTTAATCTGAACGC
CCGAAAACGCTGAAATGTGTAAAACAAACCGCCGCTAATGGTACTTTCCCCCAGCCGATATCACTGAA
AATGTGTTACTTTTGGACGCATAATTAATTTCTGATTGAGCCCTGAAGCAGCTCGACAATATTACGCATT
B12

GGTTTTTTCCCTCCCTCCTCAATCTGCTGAGTTATTTTTTATGCATATCCATTCACAATATAAGTCTATATT
TTTCTCTTCGCATTTGCCCTCGATAAAAAGCAAAATAATAATGAATCATTTCATAAATAATGGGTTTAGG
GGCTTATCGACC**GCGCAGTGGCCGCCACGGCGCTCTTATCTCGCCTGGCCACATTGCGCCCGTG**
TTCCGCTCCCCATACTGAGAGGCGCGCGCTGGACGTGATGTGGAATTATATAGACCTCACTTCC
B13

AGTTCCACACAGCACTTCAGTGCATCTAAAACCTCAGACATGGATGACCCTCCACAAAATGGCTCCAT
GCACACACTAACATCCACCGCCCGACGACGGTAAGAGGCGAATATTGGTGTTTCTGCGCTATTTTGATA
CTAACGCAGATCATAATTAGGCGTTCGATCGACGCGAGATGATTAATATGCGGTCATGTTGGGTTTATG
B14

GCATTTTTATTTGGCTGTATTCGGTCCTGGAGCATATAGGCCCTTGAGCCTGCAGAATACAGAAATCGAT
TTGGCATTTCATTTCTAATTAAAGAGCATTTTTGACATGATTTTTCCTATCTAAAAGTTAGGAAATTCGTT
B15

TCGATTTTCCATTAAAAACATCCAGGCCCGCTATTTTTTTTTCGTTTTGATAATAGACATATAAACTCATA
ACAATCCTAATAATAATATTCTTATAGGATTCTTCATTTCTAAAACTAAAATGGACATTTATATTTTAGCTA
GGCTACTATACAAGTCGGATATGATTTATTTGTATTTTTGAAATGAGTTGTCATTCACGTGTCGAATTTAG
TGTCTAATTGCGACCTTAAATATTGTTTTATCCCCGTTAAATCTGCTCGTTTCATGTCTGAATCAAAACG
TTTTGTTTTAACAACTCACAACATTTGCTAAAATCTAAATGTGCAATACTATTTCTTATTAAATAACTATA
CTGAGTAAACAAGACTGCAGGCTACAATTAAACAGTCTAAATTGCGTAAATGCCTCCATGATATTTTGC
B16

AGCTTGTTCTGTGTAAATACAAATAGGGTAATTATTAATGCATTAATAAATAAGTGGCACTATATTTACAC
B17

ACAAACAAATTGGCAAAATATTGCCAAGACTCATAGATGGAAGAGGCTGAAAACGATACATAAAAACT
AGGCTACATATATATAACGTGCATCACATCACAACGGATTGCACCGACGATATTATTAAAGACATAAACA
GGCAAATTAACAAGCCAACTGTTTTTGTAAGATTTACCATCTGAAATAAAAAGCAATGACATTAAAAA
B18

AGTGCAACAAATAAATAAAACGCAACATAATCTCATCTTAAAGTCTGGATTAAATAAAAGATGTAAATA
CAACGCCATAAACGAAGCTTCTGTTTTGCTTATAAAAAAAAAAAAAACATTAGAAACTAATAAATAGAA
ATAGGATATTTACACTGACATGATATAATTTGTTTACATTGGCGAGCAATGCCAAAAATAATAATAATAA
B19 B20

TTACGTTTATTTGACATTGAAATACGGTTTAATATGCCTATCTGGGTATGTGTTATTGAATCATCCTGTAA
TAATTATGCAGATCATATTCTGTTATAAATGTACAACCATGTTAGCAGAAGATAAGCTCTGTCCCAACGA
B21

AGAAACAGGCTTATCTCCAGATACCATGAGCAGAGCAGTGACAAACCAAGGTCAGGTCGAGACATAA
AACCACACCACAAACATGTAGAACTCCTAAAAAAATTCAACAGAGTCAACAGATGAACCATTTTTGGT
TTCTCGAGGAACTATCCCTAAAAAAAACATCCATTTATTTCTTGAATGAAGAACATTTCAATAACAAAG
ATGTTTTTCCAGTTTAAAGAATGTAAATGATCCATATATGTCCTCAGTGGAGTCACCGACATCAATCCTT
TAAATTTTAAAGTGTCCAACCTTCCTGTTATTGAAAGCAAAAGCGCGTAGAACTGATCTCACAGGCTTT
TTATATGGATACAGCAGCAGACACATATGACACGTGCTGTCATAGAGCTTCATTACTGTACTGTCAGTGT
TTCCAGTCACGCGCGCGCCGTGGACAGAAAGGATGCGCGTTAAAAAAGCTCCTTTCCTCCACTCACG
CACGCGTTCATAAAGGTGTGTTCTTTCACAGGTGACCTCGGATGAGAAGCTAAACCGGGATTTACAAC
TTGATTTGGATACCACTTTGTACATTTTCTGGGATCGCGCCGAAGG***ATG***

Figure S7. Potential binding sites of Vsx1 at proximal promoter of *tal1* (B_1-B_{21}). Yellow region indicates GC-rich region. The putative core element of promoter is in bold. The 5'-UTR and the intron within the 5'-UTR are in red and green, respectively. The start codon is in italics.

Supplementary Table S1: Primers used for synthesis of RNA probes.The restriction enzyme sites are underlined.

primer name	Sequence（5'-3'）
ZF-*tal1*-ORF-S	**CGCCGGAATTCATGATGGAAAAACTGAAATCCGA**
ZF-*tal1*-ORF-AS	**TTATCTCGAGAGGGCCTGGACTCCACTGATGAGT**
ZF-*vsx2*-ORF-S	**TAGAATTCATGACAGGAAAGGATGGGGCTGTTTT**
ZF-*vsx2*-ORF-AS	**TCTCGAGGCTCTTTTCCTCTTCTTTTGCGTCTAT**
ZF-*eng1b*-ORF-S	**CGGAATTCATGGACGAGCAAAAGGATCAAAATAG**
ZF-*eng1b*-ORF-AS	**GCTCGAGTTATTCGCTATCTTCTTTTTCGTCTTG**
ZF-*islet1*-ORF-S	**CTGAATTCATGGGAGACATGGGGGATCCAC**
ZF-*islet1*-ORF-AS	**ATCTCGAGCTAGGCCTCTATAGGACTCGCTACCA**
ZF-*islet2*-ORF-S	**CTGAATTCATGGTGGATATTCTACCGCATC**
ZF-*islet2*-ORF-AS	**TATCTCGAGTCACGTCTCCACGGGACT**
ZF-*foxn4*-ORF-S	**ATGCGAATTCTGTCCTTGAACAAGTGCT**
ZF-*foxn4*-ORF-AS	**GCGCTCGAGTTATAGCAGAACGATAGGTT**
ZF-*slc17a6b*-ORF-S	**ACTGAATTCTTCGTGCGACTGTACT**
ZF-*slc17a6b*-ORF-AS	**TACTCGAGAATAATTTCCACCCTGC**
ZF-*gad1b*-ORF-S	**CACTGAATTCGGTTGCGCGGTATAA**
ZF-*gad1b*-ORF-AS	**ATCTCGAGCTTCGTTAAAAGGGTGC**
ZF-*vsx1*-ORF-S	**ATCGGAATTCGCCACCATGACGGGAAGAGA**
ZF-*vsx1*-ORF-AS	**GGCCTCGAGAGGTCCATTTTTAACTCTCATTTTC**
ZF-*sox1a*-ORF-S	**ACGAATTCATAGCATGATGATGGAAACGGACCT**
ZF-*sox1a*-ORF-AS	**TACTCGAGCAAATATGCGTCAGTGGGACAGTTC**
ZF-*sox1b*-ORF-S	**ACGAATTCATGTATAGCATGATGATGGAGACGG**
ZF-*sox1b*-ORF-AS	**ATGGTACCCATATGTGTGTCAGTGGAACGGTTC**

Supplementary Table S2: Primers used for ChIP assay

Region	Primer name	Primer sequence （5'-3'）
region1	CHIP-s1	TAGCAATGAACTAACAAAGGCCCGAGATCAAA
	CHIP-as1	GTTGACTGACTACTGAAGATGTTTCCCTGATG
region2	CHIP-s2	CCATCATCAGGGAAACATCTTCAGTAGTCAGT
	CHIP-as2	ATGTCATTGGCCCATGAACATTTCCTGCTTGT
region3	CHIP-s3	AACAAGCAGGAAATGTTCATGGGCCAATGACA
	CHIP-as3	AACCTCCTTGCTGTGAGTTGCGATTGTGCTAT
region4	CHIP-s4	AGGTGCTGGATAAGTTGGTTCATTCCGCTGTG
	CHIP-as4	TAAGGTAAGCAGACAAACGCCAACCCAAGAAT
region5	CHIP-s5	TGCATGTATGGAGAGCAGGGTAAA
	CHIP-as5	TAAGGTAAGCAGACAAACGCCAAC
region6	CHIP-s6	TTGGCGTTTGTCTGCTTACCTTAATAACCTGC
	CHIP-as6	CGAGCTGCTTCAGGGCTCAATCAGA
region7	CHIP-s7	ATTTCTGATTGAGCCCTGAAGCAG
	CHIP-as7	CATTTTGTGGAGGGTCATCCATGT
region8	CHIP-s8	ACCTCAGACATGGATGACCCTCCACAAA
	CHIP-as8	TGCTCCAGGACCGAATACAGCCAAATAA
region9	CHIP-s9	TATTTGGCTGTATTCGGTCCTGGAGCAT
	CHIP-as9	GTCGCAAATAGACTCTAAATTCGACACG
region10	CHIP-s10	CCCCGTTAAATCTGCTCGTTTCATGTCT
	CHIP-as10	CGTTGTGATGTGATGTGATGCACGTTAT
region11	CHIP-s11	CCAAGACTCATAGATGGAAGAGGCTGAA
	CHIP-as11	GACTTTAAGCTGAGATTACGTTGCGTTT
region12	CHIP-s12	AAACGCAACGTAATCTCAGCTTAAAGTC
	CHIP-as12	TTTCTTCGTTGGGACAGAGCTTATCTTC
